# Identifying network structure similarity using spectral graph theory

**DOI:** 10.1007/s41109-017-0042-3

**Published:** 2018-01-31

**Authors:** Ralucca Gera, L. Alonso, Brian Crawford, Jeffrey House, J. A. Mendez-Bermudez, Thomas Knuth, Ryan Miller

**Affiliations:** 1 Instituto de Física, Benemérita Universidad Autónoma de Puebla, Apartado Postal J-48, Puebla, 72570 Mexico; 2 Department of Applied Mathematics, 1 University Avenue, Naval Postgraduate School, Monterey, 93943 CA USA; 3 Department of Computer Science, 1 University Avenue, Naval Postgraduate School, Monterey, 93943 CA USA; 4 Department of Operation Research, 1 University Avenue, Naval Postgraduate School, Monterey, 93943 CA USA

**Keywords:** Network topology, Graph comparison metrics, Laplacian, Eigenvalue distribution, Kolmogorov-Smirnov test

## Abstract

Most real networks are too large or they are not available for real time analysis. Therefore, in practice, decisions are made based on partial information about the ground truth network. It is of great interest to have metrics to determine if an inferred network (the partial information network) is similar to the ground truth. In this paper we develop a test for similarity between the inferred and the true network. Our research utilizes a network visualization tool, which systematically discovers a network, producing a sequence of snapshots of the network. We introduce and test our metric on the consecutive snapshots of a network, and against the ground truth.

To test the scalability of our metric we use a random matrix theory approach while discovering Erdös-Rényi graphs. This scaling analysis allows us to make predictions about the performance of the discovery process.

## Introduction

The successful discovery of a network/graph is of great interest to the Network Sciences community. Many algorithms have been proposed for network discovery. But, our focus is on when have we discovered enough of the network to be similar to the ground truth, namely representative of the whole network. We measure similarity of temporal snapshots of a network, as it is discovered through monitor placement, by comparing consecutive temporal snapshot (subgraphs) produced in the inference of the network.

For a given network, one perspective on network discovery is to consider any subgraph as one of many possible outcomes from some discovery process. For a simple graph *G*(*V,E*), with |*V*(*G*)|=*n*, and |*E*(*G*)|=*m*, there are 2^*m*^ possible subgraphs on *n* vertices. In real-world applications, say if *m*=1200, the count of possible subgraphs grows rapidly: 2^1200^ is on the order of 10^360^. Any discovered subgraph is one of many possible random outcomes. We wish to determine whether one collection of discovered nodes and edges is very similar to the underlying graph.

A comparison technique like percent of vertices discovered, percent of nodes discovered, or degree sequence distribution reveals a practical problem. In general, we do not know what the underlying network looks like. How do we compare graphs to the ground truth if we do not know the ground truth? We search for a method that identifies similar graphs during the discovery process, so that we know at what point of the inference only little information is being discovered, so pursuing the inference has little benefit.

The method we use is based on nonparametric statistical tests, that tells if two consecutive snapshots are similar, without actually knowing the true network. In a two sample nonparametric test we compare two samples and assume they came from the same distribution. The alternative hypothesis is that there was significant change between the two samples. For this purpose, we introduced in Crawford et al. (2016), the two sample nonparametric test on Sequential Adjacency and Laplacian Matrix Eigenvalue Distribution. For a proof of concept, we examined a synthetic network (an Erdös-Rényi random graph) and three terrorist networks.

In the current paper, for a complementary analysis, we also use normalized Laplacian Matrix Eigenvalue Distribution to compare snapshots. We contrast the methodology based on either the Sequential Adjacency, or Laplacian or normalized Laplacian Matrix Eigenvalue Distribution. Furthermore, we perform a theoretical systematic study of our metric while discovering ensembles of Erdös-Rényi graphs. This allows us to make predictions about the performance of a discovery process, characterized by our metric, once the basic properties of a network (of Erdös-Rényi–type) are known. We then present the resulting theory on a terrorist network for validation.

## Background

In graph theory, an established metric for graph comparison is isomorphism. Two labeled graphs *G* and *H* are *isomorphic* if there exists a bijection *ϕ* from *V*(*G*) to *V*(*H*) such that *uv* ∈*E*(*G*) if and only if *ϕ*(*u*)*ϕ*(*v*)∈*E*(*H*) (Chartrand and Zhang 2012). Comparing graphs based on isomorphism has a binary outcome: the graphs are either exactly the same (isomorphic), or they are different (non-isomorphic). In practice we prefer similarity values to belong to a range, and to converge as we approach isomorphism. We build in the validation of our comparison methodology by using a network discovery process (or lighting up a network) that produces a sequence of consecutive temporal snapshots. An assumption we make is that consecutive snapshots of the network are similar, which was validated using http://faculty.nps.edu/rgera/projects.html (Gera 2015).

### Similarity of networks

Existing similar research has considered the count or the percent of nodes/edges discovered during a network’s discovery. For a network *G*, this was done by measuring the percent that has been discovered at step *i* with the subgraph *G*_*i*_ through tracking |*V*(*G*_*i*_)|/|*V*(*G*)| and |*E*(*G*_*i*_)|/|*E*(*G*)|. While both are great intuitive measures, they only captures the cardinality of sets of nodes and edges discovered, but not so much the topology of the network itself (Davis et al. 2016; Chen et al. 2017; Wijegunawardana et al. 2017).

Common metrics for measuring general network similarity use comparison of degree distributions, density, clustering coefficient, average path length and etc. Inexact matching of two networks after a sequence of edits is commonly measured by *Graph Edit Distance* (GED). GED measures the cost of adding/removing/substituting nodes and edges to make one graph look like the other one. This works well for shortest paths rather than arbitrary graphs. Algorithms use combinatorial searches over the space of possible edits, therefore they are computationally intensive.

To optimize this idea, *Kernel functions* are used that explore regions of the network to be matched using GED such as:

- *Node/Edge Kernel:* For labeled graphs, whenever two nodes/edges have the same label, the kernel function value is 1, otherwise 0. If the node/edge labels take real values, then a Gaussian kernel is used.
- *Path kernel:* Whenever two paths (sequences of alternating nodes and edges) are of the same length, the path kernel is the product of the kernels of the node and edge on the paths. If the length of the common paths is different (i.e., the algorithm didn’t detect a common path between the networks), the value of the path kernel function is 0.
- *Graph kernel:* The graph kernel compares subgraphs of each of the graphs by comparing the proportion of all common paths out of all possible paths in each of the two subgraphs.

Kashima and Inokuchi (2002) map networks to feature vectors and then cluster the vectors based on Naïve distance methods. Attributes about the data are needed in order to create the feature vectors, which they mine using search engines on the Web.

More sophisticated methods consider capturing networks’ topology before comparing them. For example, Gromov–Hausdorff (G-H) distance uses shape analysis. The network’s shape is constructed by piecing together small subgraphs whose similar structure is easy to find. This shape is then transformed into a linkage matrix that captures how these subgraphs interconnect. Then the G-H distance is the farthest distance any node of a network *G* is from the network *H*, or the farthest any node of *H* is from *G*, whichever is greater, taken over all possible embedding (drawing) of the two networks *G* and *H* (Lee et al. 2011).

Similarly, Pržulj uses graphlets (Pržulj 2007) to capture the topology of a network. Graphlets are all possible subgraphs of small number of nodes capturing the local structure properties. Graphlet Frequency Distribution can be used to compare networks, by keeping track of the frequencies of all the different size graphlets (Rahman et al. 2014). This is unfeasible for large graphs as it requires an exact count of each graphlet. It has been used in comparing aerial images (Zhang et al. 2013), scene classification plays an important role in multimedia information (Zhang et al. 2011), and learning on synthetic networks (Janssen et al. 2012).

Koutra et al. proposed DeltaCon (Koutra et al. 2013), a scalable algorithm for same size networks, based on nodes influence. It compares the two networks with the exact same node set, by computing each node’s influence on the other network’s nodes. These values are stored as matrices for each network, and the difference between the matrices is then measured to give an affinity score measuring similarity.

Feasible inexact matching of different size graphs use spectral analysis. The spectrum of classes of graphs has been questioned since 1956 (Günthard and Primas 1956), and further studied since then (VanDam and Haemers 2003; Florkowski 2008; Gera and Stanica 2011). General graph theoretical results are known since the 70*s* (Cvetković 1971), reviewed in (Chung 1997), and later extended for complex networks (VanMieghem 2010). Spectral clustering of graphs can use the eigenvalues of several matrices (Wilson and Zhu 2008). We use the *adjacency* matrix *A*, the *Laplacian* *L*=*D*−*A* (where *D* is the degree matrix), and *normalized Laplacian* defined as the matrix:

$$L(u,v) = \left\{ \begin{array}{ll} 1 & \quad \text{if} \ u=v \ \text{and} \ d_{v} \ \neq 0,\\ -1/\sqrt{d_{v} \cdot d_{u}} & \quad \text{if } u \neq v, \text{ and } u \text{ adjacent to } v,\\ 0 & \quad \text{otherwise,} \end{array}\right. $$

where *d*_*v*_ denotes the degree of the vertex *v*.

The eigenvalues of each of these matrices define the spectrum of the network. While the existence of cospectral graphs (i.e. graphs that share the same adjacency matrix spectrum) is known since 1971 (VonCollatz and Sinogowitz 1957; Harary et al. 1971) and ongoing research considers the graphs that are determined by their spectra (VanDam and Haemers 2003), spectral analysis is very useful in comparing networks as we explain next. Particularly, since finding these co-spectral graphs is “out of reach” (Schwenk 1973; Godsil and McKay 1982).

Eigenvalue analysis is used to describe the behavior of a dynamic system (Trefethen and Bau III 1997), and in our case, the behavior of a network representing the system. To see its relevance in comparing networks, note that eigenvalues measure the node cluster cohesiveness or community structure that has widely been studied in network science. Moreover, the algebraic connectivity of the graph, and thus the spectra, captures the topology of the graph (Frankl and Rödl 1987). Of particular interest for us, is that the spectral clustering can differentiate between the structural equivalence and the regular equivalence of nodes. For the structural equivalence, nodes are placed in the same community if they have similar connection patterns to the *same* neighbors. For the regular equivalence, nodes are placed in the same community if they have similar connection patter to *any* neighbors. Of course, this can be extended to probabilistic models where stochastic equivalences are introduced based on groups being stochastically equivalent if their respective connecting probabilities to neighbors are the same.

The distribution of eigenvalues of the adjacency matrix can be found in Chung (1997), focused on the correlation of the range of the distribution of eigenvalues to the type of graph. This was further studied as the behavior of the distribution of the eigenvalues of the graph, such as convergence results in (Dumitriu and Pal 2012). Correlations between the power law distribution of the graph and the distribution of the eigenvalues have been presented in (Mihail and Papadimitriou 2002). Analyzing several real graphs, they inferred that if the degrees of the graph *d*_1_…*d*_*n*_ were power law distributed, then there is a high probability that the eigenvalues of the graph will be power law distributed (and take on the values $\sqrt {d_{1}}\ldots \sqrt {d_{n}}$) (Mihail and Papadimitriou 2002).

The distribution of the eigenvalues of the Laplacian is more closely linked to the structure of the graph than only using the eigenvalues of the adjacency list (Chung 1997). The *normalized* Laplacian contains the degree distribution as well as the adjacency matrix information from the graph. While spectral analysis was previously used to cluster similar trees and synthetic graphs (Wilson and Zhu 2008), we use the spectra within a different methodology based on nonparametric statistics.

Nonparametric statistical tests can capture whether two samples of a network are similar without actually knowing the true networks. We compare the eigenvalues distributions of two samples (subgraphs) and test the assumption they came from the same distribution. The alternative hypothesis is there is significant change between the two samples. By looking at the actual step by step inference using Gera (2015), we could visually see small differences between consecutive snapshots. No major changes happen in consecutive snapshots.

We use the nonparametric test of Ruth and Koyak (2011), where the first *m* of *N* observations *X*_1_⋯*X*_*m*_⋯*X*_*N*_ are assumed to follow distribution *F*_1_ and the rest are from *F*_2_. This allows us to see a “shift point" at *X*_*m*+1_ where our samples are no longer from the same distribution. For our research, each observation *X*_*i*_ is the eigenvalue distribution of a sampled/inferred graph. In our case, the null hypothesis is the the distributions of eigenvalues of the two samples are the same. Each test will yield a *p*-value representing the probability that the test statistic would be as extreme or more extreme than what was observed with a particular sample, assuming the null hypothesis is true. If two samples have very different eigenvalue distributions, the null hypothesis is less plausible and the *p*-value is low. If two distributions of eigenvalues match, that is strong evidence in support of the null hypothesis, and any other outcome would be more extreme than what was observed with a have close to 1 *p*-value.

### Scaling the Erdös-Rényi networks

We will use the Erdös-Rényi random graph model for a scaling analysis of the metric. The Erdös-Rényi random graph model is characterized by two parameters: the number of nodes (or graph size) *N* and the connectivity probability *α*, where *α* is defined as the fraction of the *N*(*N*−1)/2 independent non-vanishing off-diagonal adjacency matrix elements. All the nodes are isolated when *α*=0, whereas we have a complete graph for *α*=1.

From a *random matrix theory* point of view it is a common practice to look for the scaling parameter(s) of a random matrix model; in this way the *universal properties* of the model can be revealed (Méndez-Bermúdez et al. 2017a; 2017b). Scaling studies of the Erdös-Rényi random graph model can be found in Méndez-Bermúdez et al. (2015) and Martínez-Mendoza et al. (2013). The average degree is then

1 $$ \xi = \alpha \times N,  $$

where *ξ* is the mean number of nonzero elements per adjacency matrix row, called the *scaling parameter* of the Erdös-Rényi random graph model. In particular, it was shown that spectral, eigenfunction, and transport properties are universal (i.e. equivalent) for a fixed average degree *ξ*.

In fact, several papers have been devoted to analytical and numerical studies of the Erdös-Rényi random graph model as a function of *ξ*. Among the most relevant results of these studies we can mention that: (i) In the very sparse case (*ξ*→1) the density of eigenvalues was found to deviate from the Wigner semicircle law with the appearance of singularities, around and at the band center, and tails beyond the semicircle (Rodgers and Bray 1988; Rodgers and deDominicis 1990; Mirlin and Fyodorov 1991; Evangelou and Economou 1992; Evangelou 1992; Semerjian and Cugliandolo 2002; Khorunzhy and Rodgers 1997; Kühn 2008; Rogers et al. 2008; Slanina 2011); (ii) A delocalization transition of eigenstates was found at *ξ*≈1.4, see Mirlin and Fyodorov (1991); Evangelou and Economou (1992); Evangelou (1992) and Fyodorov and Mirlin (1991); (iii) The nearest-neighbor eigenvalue spacing distribution *P*(*s*) was found to evolve from the Poisson to the Gaussian Orthogonal Ensemble (GOE) predictions for increasing *ξ*, see Jackson et al. (2001); Evangelou and Economou (1992) and Evangelou (1992) (the same transition was reported for the spectral number variance in Jackson et al. (2001)); (iv) The onset of the GOE limit for the spectral properties occurs at *ξ*≈7, see Méndez-Bermúdez et al. (2015) and Martínez-Mendoza et al. (2013), meaning that the spectral properties of the graph above this value coincide with those of a system with maximal disorder. Also, the first eigenvalue/eigenfunction problem was addressed in Kabashima et al. (2010) and Kabashima and Takahashi (2012).

For our paper, following Méndez-Bermúdez et al. (2015) and Martínez-Mendoza et al. (2013), we look for universal properties of the discovery algorithm.

## Methodology

Using the Network Visualization Tool (Gera 2015), we choose a discovery algorithm and run it on a network. This produces the sequence of inferred subgraphs to be analyzed for comparison. The chosen algorithm is not relevant, it merely creates the sequence of subgraphs. For our research, we chose Fake Degree Discovery, a sophisticated degree greedy algorithm (Gera et al. 2017) (code is available at https://github.com/Pelonza/Graph_Inference/blob/master/Clean_Algorithms/FDD.py, see Schmitt (2015), and it can be tested at http://faculty.nps.edu/rgera/projects.html (Gera 2015)).

Let *G*_*i*_ be a sequence of graphs recorded while lighting up some given graph *G*, where, if *i*<*j*, then *G*_*i*_ was discovered before *G*_*j*_, and *G*_*i*_⊆*G*_*j*_, ∀*i*≤*j*. For each of the adjacency, Laplacian, and normalized Laplacian matrix let *Λ*_*i*_ be the list (or vector) of ordered eigenvalues for *G*_*i*_. Also, let *Λ* be the vector of eigenvalues from the (true) underlying graph *G*. Note these are not eigenvectors - each is a vector of eigenvalues (of the adjacency or Laplacian or normalized Laplacian matrices). Then if *G*_*i*_=*G*, it follows *Λ*_*i*_=*Λ*. During the process of discovering the network, we will not achieve *Λ*_*i*_=*Λ*, but we expect that *Λ*_*i*_→*Λ* as *i* increases.

We then apply the nonparametric test, to compare a sample of data to a known distribution and measure the “goodness of fit.” We test the null hypothesis *Λ*_*i*_=*Λ*_*j*_ for *i*<*j*. For large *i* and *j* values, we expect that when the difference between *i* and *j* is small, that we would fail to reject this hypothesis. This leads to the conclusion that the subgraphs are similar at point *i* and *j*. Note that failure to reject the null hypothesis does not imply the hypothesis is explicitly true. Rather, it means we have no evidence that it is false. Thus we should not conclude *Λ*_*i*_=*Λ* when we fail to reject the null hypothesis.

## Results and analysis

In Crawford et al. (2016), we analyzed test-cases using our algorithm on a randomly generated Erdös-Rényi graph, and three real terrorist networks. Each of these networks were discovered using Fake Degree Discovery. The analysis of each of these networks included the distribution of eigenvalues from the adjacency matrix and the Laplacian. The difference in distributions was observed: A subgraph from early in the process of network discovery has a different eigenvalue distribution from the true underlying graph. Yet as network discovery progresses, this difference in distributions decreases. This difference was more pronounced when the eigenvalues of the Laplacian matrix were used.

For the current analysis, we present (1) an experiment on a real network extending the analysis in Crawford et al. (2016), (2) a theoretical scaling analysis of the discovery algorithm on ensembles of Erdös-Rényi random graphs, and (3) a comparison between the two sets of networks (synthetic and real-world) by applying the main result of the scaling analysis to the Noordin Top’s network.

### Preliminary experiment

We augment the analysis in Crawford et al. (2016) by considering the normalized Laplacian’s eigenvalue distribution. As an example, we present one of the terrorist networks, as it works similarly for the others. Noordin Top terrorist network is the aggregation of 14 different relationship types amongst 139 terrorists for a total of 1499 edges. This network captures the relationships of five major terrorist organizations that operate in Indonesia. Noordin Top is the key broker between these organizations and exercises his influence to conduct large scale terrorist training events and operations.

**Fig. 1:**
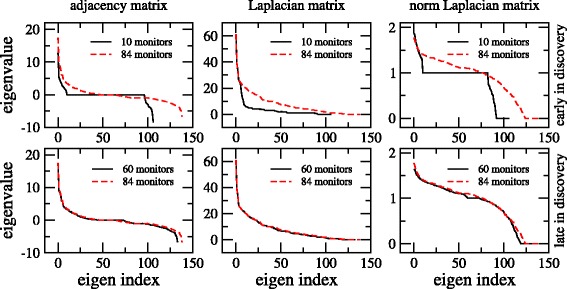
Comparison of eigenvalue distributions at step 10 (*upper* panels) and step 60 (*lower* panels) against ground truth (*red-dashed lines*) for adjacency, Laplacian, and normalized Laplacian matrices of the Noordin Top terrorist network

Partial information graphs were captured at step 10 as an early discovery, and step 60 as late in discovery, out of 84 possible steps considered the terminal discovery step shown in Fig. 1.

The normalized Laplacian matrices generated on the Noordin Top terrorist network are displayed in Fig. 1 (right panels), which are new to our analysis. We can visually see a larger dissimilarity, both in the early and late steps compared to adjacency and Laplacian distributions of Fig. 1 (left and middle panels). Notice the progressive convergence towards the eigenvalue distribution in the final step, for all three matrices.

**Fig. 2:**
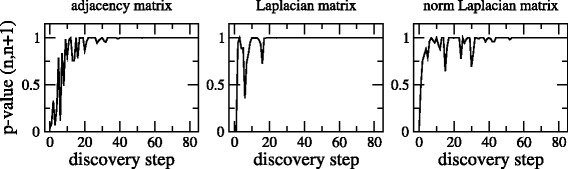
Sequential nonparametric test *p*-value as a function of discovery step for adjacency, Laplacian, and normalized Laplacian matrices of the Noordin Top terrorist network

To formally measure these differences in distributions we present the nonparametric test results in Fig. 2. In practice, in contrast to the Network Visualization Tool (Gera 2015), network discovery is a sequential process and the true underlying graph is not available for comparison. Therefore we do not have the luxury to compare against ground truth. In Crawford et al. (2016), we determined that the nonparametric test is also useful when comparing sequential snapshots of a network.

Figure 2 shows that the normalized Laplacian’s *p*-values are consistent with what was discovered for the adjacency matrix and the Laplacian: monitors placed during the early inference stages bring relatively many vertices and edges, than they do later in the discovery, relative to the size of the graph. This makes the newly discovered graph be more similar to its predecessor in the late discovery. When a monitor finds little new information, the nonparametric test has a high *p*-value for consecutive snapshots (meaning that they are similar) and it eventually stabilizes remaining high.

One interpretation of our results is that we have discovered the “essential elements” of the graph about the time the values of *p* stabilizes. We base this statement on the rapid climb in the *p*-value for the nonparametric test that occurs in the beginning, stabilizing after a point. Since this plot shows the *p*-value when comparing sequential steps of the inferred graph, the steep climb in *p*-value represents a transition zone - from having dissimilar consecutive snapshots based on the amount of information discovered, to similar consecutive snapshots towards the end of the inference.

We find this same very steep transition occurs much later for the normalized Laplacian. This is consistent with distribution of eigenvalues from the Laplacian, including characterizations of graphs based solely on normalized eigenvalues. The Laplacian eigenvalue distribution comparison method is slower to conclude graphs are similar because it is armed with more information, so it is more sensitive to small changes.

The rapid stabilization of the nonparametric test when comparing consecutive snapshots is useful when comparing graphs in the setting where the true underlying graph remains unknown or unknowable. The advantage of such a metric is that it is self-referential: Nothing needs to be assumed beyond what has been discovered. The desirable property of early stabilization can be put to use when it fails: After the nonparametric test measure stabilizes and discovery continues, a break in stability marks a major discovery.

We now proceed with the theoretical analysis by scaling the networks.

### Scaling analysis

We now look for the universal properties of the discovery algorithm, characterized by our metric, when applied to Erdös-Rényi graphs. For this purpose we choose curves of the nonparametric test *p*-value (against ground truth) as a function of the discovery step.

**Fig. 3:**
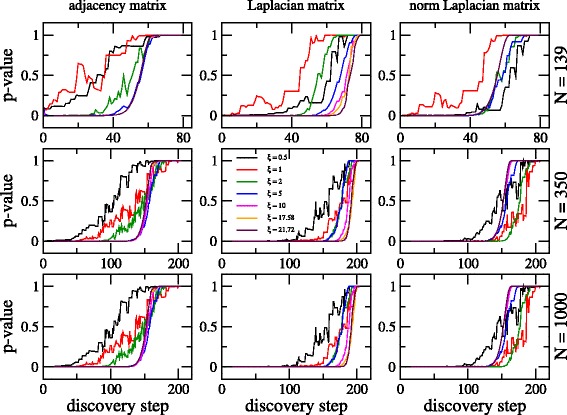
Nonparametric test *p*-value (against ground truth) as a function of discovery step for adjacency, Laplacian, and normalized Laplacian matrices of Erdös-Rényi graphs. Graphs of size *N*=139 (*upper* panels), *N*=350 (*middle* panels), and *N*=1000 (*lower* panels). Several values of *ξ* are considered

As a starting point in Fig. 3 we present curves for the nonparametric test *p*-value as a function of discovery step for adjacency, Laplacian, and normalized Laplacian matrices of Erdös-Rényi graphs for increasing average degree *ξ*. In this figure we present results for three graph sizes: *N*=139 (the number of nodes of the Noordin Top terrorist network), *N*=350 (a larger graph of the same size as the one in Crawford et al. (2016)), and *N*=1000 (an even larger graph case).

Note that we are including curves for *ξ*=17.58 and 21.72, the values of the average degrees of the Erdös-Rényi graph used as example in Crawford et al. (2016) and of the Noordin Top network, respectively. From this figure we observe that: (i) For small average degree, i.e. *ξ*≤5, the curves of nonparametric test *p*-value vs. discovery step show strong fluctuations, moreover, the curves change significantly by changing the value of *ξ*; (ii) For large enough *ξ*, i.e. *ξ*≥10, the curves of nonparametric test *p*-value vs. discovery step become smooth and do not change much by further increasing *ξ*. We understand this by recalling that the onset of the GOE limit for Erdös-Rényi graphs occurs at *ξ*≈7 (Méndez-Bermúdez et al. 2015; Martínez-Mendoza et al. 2013). This means that since the properties of the graph above this value coincide with those of a system with maximal disorder (the most complex scenario) they do not evolve further by increasing *ξ*.

**Fig. 4:**
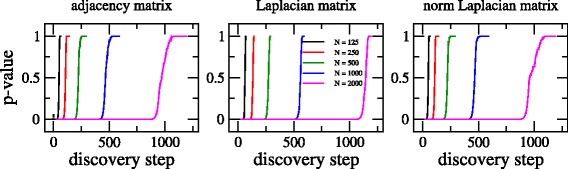
Nonparametric test *p*-value (against ground truth) as a function of discovery step for adjacency, Laplacian, and normalized Laplacian matrices of Erdös-Rényi graphs. Several graph sizes *N* are considered. *ξ*=10 in all cases

It is also instructive to look at the curves of the nonparametric test *p*-value as a function of discovery step for increasing graph size at fixed average degree. This is done in Fig. 4 where we show curves for adjacency, Laplacian, and normalized Laplacian matrices of Erdös-Rényi graphs having *N*=125, 250, 500, 1000, and 2000. We have fixed the value of *ξ* to 10, i.e. already above the onset of the GOE limit. It is evident that these curves are displaced to the right for increasing *N*.

**Fig. 5:**
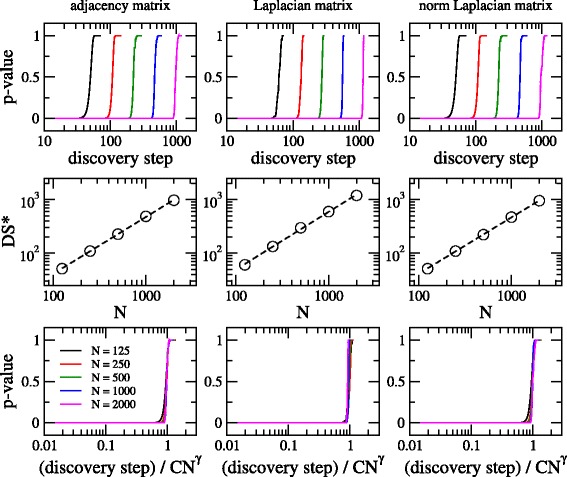
(*Upper* panels) Nonparametric test *p*-value as a function of discovery step for adjacency, Laplacian, and normalized Laplacian matrices of Erdös-Rényi graphs of increasing sizes (same curves as in Fig. 4). (*Middle* panels) DS* as a function of *N* (symbols) and fitting of the data with Eq. (2) (*dashed lines*). The values of the fitting parameters are reported in Table 1. (*Lower* panels) Same curves as in upper panels but with the discovery step divided by ${\mathcal {C}} N^{\gamma }$. *ξ*=10 in all cases

Moreover by plotting the curves of Fig. 4 now in semi-log scale (see upper panels of Fig. 5), it is clear that the displacement to the right on the *x*-axis looks constant by duplicating the graph size *N*. This observation made us think that these curves may accept a scaling (somehow dependent on *N*) on the discovery step.

**Table 1:** Values of parameters $\mathcal {C}$ and *γ* from the fittings of the data of Fig. 5 (middle panels) with Eq. (2)

	Adjacency	Laplacian	Normalized Laplacian
$\mathcal {C}$	0.309±0.056	0.349±0.133	0.324±0.046
*γ*	1.062±0.009	1.075±0.021	1.052±0.007

To look for that scaling we first define a quantity that can characterize the displacement of the curves produced by changing *N*. We note that all curves in the semi-log scale look very similar: They are approximately zero for small discovery step, then grow, and finally stabilize to 1. So, we can choose as the characteristic quantity, for example, the discovery step value at which the curves start to grow, the value at which the curves approach one, or the value at which the curve derivative reaches its maximum in the transition region. We choose the last quantity that we call DS*.

In the middle panels of Fig. 5 we present DS* as a function of *N* in log-log scale. The linear trend of the data (in the log-log scale) implies a power-law relation of the form

2 $$ \text{DS*} = {\mathcal{C}} N^{\gamma} \ .   $$

Indeed, Eq. (2) provides very good fittings of the data with values of *γ* very close to unity (reported in Table 1). Therefore, by plotting again the nonparametric test *p*-value now as a function of the discovery step divided by ${\mathcal {C}} N^{\gamma }$, as shown in the lower panels of Fig. 5, we observe that curves for different graph sizes *N* fall on top of a *universal curve*.

Finally, it is important to stress that: (i) Our scaling is expected to work for graphs of Erdös-Rényi–type only, since other random graph models may display different scaling laws, if any. (ii) Our scaling is expected to be similar for any *ξ*>7, i.e. once the Erdös-Rényi random graph model is in the maximal disorder limit. (iii) In Fig. 5 (lower panels) there is an evident finite size effect for small *N*, meaning that the *universal curve* can be defined once *N*≫1; roughly speaking for *N*>500. (iv) We do not observe any relevant difference in the scaling of the nonparametric test *p*-value curves for adjacency and normalized Laplacian matrices. (v) The onset of the discovery transition takes place at

3 $$ (\text{discovery \ step}) / {\mathcal{C}} N^{\gamma} \approx 1 \ .   $$

Moreover, the scaling shown in Fig. 5 (lower panels) can be used to predict how efficient a discovery algorithm, characterized by our graph comparison metric, will be once the average degree of the graph and its size are known: Eq. (3) means (for an Erdös-Rényi–type graph of size *N* and *ξ*>7) that a discovery algorithm needs more than ${\mathcal {C}} N^{\gamma }$ discovery steps to uncover most of the graph. See an application in “Application of the scaling analysis” subsection.

### Application of the scaling analysis

We now use the main result of our scaling analysis, i.e. Eq. (3), to estimate the performance of the discovery algorithm on the Noordin Top terrorist network (even though, this real-world network is different to the Erdös-Rényi random network model used to derive the scaling).

We first recognize that the average degree of the Noordin Top terrorist network is 21.72; well above the requirement of *ξ*>7 for the scaling to be valid. Then, with *N*=139, Eq. (3) (in combination with the values of $\mathcal {C}$ and *γ* reported in Table 1) predicts that the discovery algorithm needs more than 58 steps to uncover most of the graph when the algorithm is applied to adjacency or normalized Laplacian matrices; in the case of the Laplacian matrix the discovery algorithm needs more than 70 steps.

**Fig. 6:**
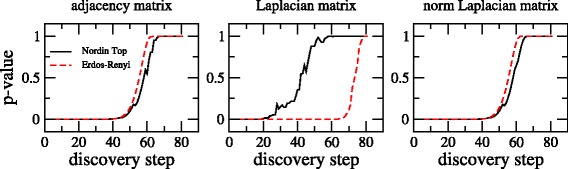
Nonparametric test *p*-value (against ground truth) as a function of discovery step for adjacency, Laplacian, and normalized Laplacian matrices of the Noordin Top terrorist network (*black curves*). *Red-dashed curves*, included as a reference, correspond to the nonparametric test *p*-value of an Erdös-Rényi graph with same number of nodes and edges

Then, in Fig. 6 we show plots of the nonparametric tests *p*-value as a function of discovery step for adjacency, Laplacian, and normalized Laplacian matrices of the Noordin Top terrorist network and of an Erdös-Rényi graph with the same number of nodes and edges.

The good correspondence of the curves of nonparametric tests *p*-value for the adjacency and normalized Laplacian matrices of the synthetic and real-world networks validates the applicability of Eq. (3): Indeed, it is clear that the discovery algorithm needs just about 58 steps to uncover both networks. However, for the case of the Laplacian matrix the discovery algorithm works much faster on the real-world network; see the middle panel in Fig. 6. Therefore, it is a good proxy even for networks that are far from being random.

## Conclusions

This paper explores the potential of eigenvalue distribution analysis for graph comparison. There are many questions this analysis could be the answer to. As stated in the introduction, there are several network discovery algorithms, and it is important to identify which algorithm is the most effective for discovering a network. The methodology developed in this research could be applied to measure the effectiveness of different types of network discovery algorithms.

We introduced a methodology that measures the similarity of networks, that we validated on consecutive snapshots of networks. We achieved it using a nonparametric test on the distribution of eigenvalues of networks, using three matrices: adjacency, Laplacian and normalized Laplacian.

Our numerical experiments show what we anticipated: using the *p*-value from the nonparametric test as a measure of similarity, (1) the distribution of eigenvalues from consecutive subgraphs become more similar as the portion of the newly discovered network is small compared to the discovered network, and (2) that the *p*-values stabilize towards the end of the discovery. Further, comparisons using this metric to the true underlying graph is a non-decreasing function of time (temporal discovery).

In addition, we performed a systematic study of our metric while discovering ensembles of Erdös-Rényi graphs. This allowed us to consider different network sizes for our analysis. The resulting scaling analysis allows us to make predictions about the performance of a discovery process that we successfully tested on a real-world network: the Noordin Top terrorist network.

We conclude that the use of sequential adjacency, Laplacian, and normalized Laplacian matrix eigenvalue distribution comparisons based on the nonparametric test *p*-values is a promising method to guide network discovery.

## Future direction

One possible extension of this paper is to explore the properties of eigenvalues from the normalized Laplacian in various graph types, particularly scale free and sparse graphs. An analysis of the properties of normalized eigenvalues from the Laplacian has led to the establishment of both specific and general boundary conditions depending on the type of graph. For example, all graphs will produce eigenvalues between 0 and 2 (Chung 1997). For a complete graph, they are bounded by 0 and *n*/(*n*−1) with multiplicity (*n*−1) (Chung 1997).

Recall that the union of two separate components results in the union of the spectrum of each component of the graph (Chung 1997). Thus, if we consider the early snapshots subgraphs in our discovery, they can be disconnected graphs. By adding more monitors, we effectively add another component to the previous subgraph. Thus the resulting updated subgraph will inherent the eigenvalues of each separate component. For example, when the eigenvalue distributions are compared, the eigenvalues of early temporal snapshot will be present in late temporal snapshot. Following this logic, it may be possible to infer missing eigenvalues from the graph of the distribution and reverse engineer the network.

Concerning the theoretical model, a next step in our research includes the scaling analysis of the discovery algorithm when applied on other graph models (as compared to the basic Erdös-Rényi random network model we use here), such as scale-free graphs. We are particularly interested in an extension to more complicated networks, such as multilayered networks, in seeing how the identification and interdependence of the layers influences our measure.
